# Design of a Web-based individual coping and alcohol-intervention program (web-ICAIP) for children of parents with alcohol problems: study protocol for a randomized controlled trial

**DOI:** 10.1186/1471-2458-12-35

**Published:** 2012-01-16

**Authors:** Tobias H Elgán, Helena Hansson, Ulla Zetterlind, Nicklas Kartengren, Håkan Leifman

**Affiliations:** 1 STAD, Stockholm Centre for Psychiatric Research and Education, Department of Clinical Neuroscience, Stockholm County Council Health Care Provision and Karolinska Institutet, Box 6031, SE-102 31 Stockholm, Sweden; 2 Clinical Health Promotion Centre, Department of Health Sciences, Lund University and Skåne University Hospital MAS, SE-205 02 Malmö, Sweden; 3 The Swedish Council for Information on Alcohol and Other Drugs (CAN), Box 704 12, SE-107 25 Stockholm, Sweden

**Keywords:** Children of alcoholics, CoA, Children of alcoholics screening test, CAST, Children of substance abusing parents, Individual Coping and Alcohol Intervention Program, ICAIP, Internet-delivered intervention, RCT, Web-based intervention

## Abstract

**Background:**

It has been estimated that approximately 20% of all Swedish children grow up with parents having alcohol problems, which may result in negative outcomes among these children. Therefore, most Swedish municipalities provide resources for support, but at the same time figures reveal that not even 2% receive support, mainly due to difficulties in identifying and recruiting these children into support programs. Delivering intervention programs to children and adolescents *via *the Internet seems a promising strategy, but to date, the number of web-based interventions aimed at this target group is very scarce. We have therefore developed a novel internet-delivered therapist assisted self-management intervention called the web-ICAIP (Individual Coping and Alcohol Intervention Program) for adolescents having parents with alcohol problems. The purpose of the program is to strengthen adolescents' coping behavior, improve their mental health, and postponing the onset or decreasing risky alcohol consumption. This paper describes the web-ICAIP and the design of a randomized controlled trial (RCT) to measure the efficacy of this intervention.

**Methods/Design:**

The RCT will include at least 183 adolescents (15-19 year old) who will be randomly allocated to two conditions where one group has access to the web-ICAIP and the other is a waiting list control group. Participants will be recruited from websites containing information and facts for adolescents about alcohol and other drugs. Possible participants will be screened using the short version of the Children of Alcoholics Screening Test (CAST-6). The assessment consists of a baseline and two follow-up measurements taking place after two and six months, respectively. The primary outcomes include the Center for Epidemiological Studies Depression Scale (CES-DC), a coping behavior scale, and also the short version of the Alcohol Use Disorders Identification Test (AUDIT-C). Additional outcomes include the "Ladder of life" which measures overall life satisfaction and questions concerning program adherence.

**Discussion:**

There is an urgent need for developing and evaluating web-based intervention programs which target children having parents with alcohol problems. This study will therefore make an important contribution to this novel field of research.

**Trial registration:**

ISRCTN41545712

## Background

There are different estimates reported on the prevalence of children having parents with alcohol problems, and these figures all indicate that the problem is wide-spread. For instance, in the U.S. it has been estimated that 28.6% of all children below the age of 18 years are exposed to alcohol abuse or dependence within the family [1], while in 15 European Union countries (and Norway) it has been estimated that between 6.8 and 11.7% of all children below 15 years of age are affected by parental alcohol misuse [2]. In Sweden, it has been estimated that approximately 20% of all children grow up in families where at least one parent has risky alcohol consumption [3].

Children affected by parental substance abuse are at increased risk for many psychological problems (reviewed in [4-6]). For instance, they have an elevated risk of developing internalizing symptoms such as depression and anxiety disorders, and externalizing symptoms such as conduct disorders [6,7]. Additionally, it has been demonstrated that these children may experience poor intellectual, cognitive, and academic achievement [8,9], domestic physical abuse [10], and are at risk of earlier drinking onset [11] and developing substance abuse problems themselves [12-14]. Children growing up in families with substance abuse problems therefore comprise a target group for selective intervention and prevention (reviewed in [15,16]).

In Sweden, the municipalities alone account for the vast majority of support offered to these children. In an annual survey from the junior association of the Swedish IOGT-NTO it is reported that about eight out of ten municipalities provide resources for support [17]. However, estimates also reveal that not even 2% of all these children receive support. Thus, there is an overwhelming majority who never receive support, mainly due to difficulties in identifying and attracting them into intervention programs [15,18].

One appealing strategy to reach out and support a larger number of people is to deliver intervention programs *via *the Internet (reviewed in [19-21]). These web-based interventions seem particularly appealing for adolescents as they generally have good computer skills and often use social media online. Furthermore, research reveal that adolescents regard the Internet as attractive since it is readily accessible and an anonymous means of seeking help [22]. The positive effects of web-based interventions have been demonstrated across a broad range of different conditions and in a comprehensive review by Barak and co-workers, covering 92 studies involving 9764 participants, an overall medium mean effects size was found, which is in line with traditional "face-to-face" therapy [23]. To date, most of these interventions have been designed for adults. However, a few interventions have been reported that target children or adolescents [24,25], but the number of web-based interventions aimed at children of substance abusing parents are still very scarce [15,26].

This paper reports on the design of a novel therapist assisted web-based self-management intervention which has its origin in a manual-based "face-to-face" intervention called the ICAIP (Individual Coping and Alcohol Intervention Program) [27,28] developed at the unit of Clinical Alcohol Research (presently named the Clinical Health Promotion Centre) at the Department of Health Sciences, Lund University and Skåne University Hospital. Previous research on both the ICAIP, aimed at college students who have parents with alcohol problems, and a coping skills intervention program, aimed at spouses of alcoholics, have shown positive effects with regard to decreased alcohol consumption, and improved mental health as well as coping behavior [27-30]. Our hypothesis is therefore that a web-based version of the ICAIP, web-ICAIP, will render in positive effects among a younger target group of 15-19 year olds who have parents with alcohol problems.

### Objective and research questions

The main objective of this study is to evaluate the efficacy of web-ICAIP which is targeted at adolescents aged between 15 and 19 years, having at least one parent with alcohol problems. Specific research questions concerns the participants' improvement in (i) coping behavior, (ii) mental health, and also relates to (iii) alcohol in the sense of postponing the onset or decreasing risky consumption.

## Methods/Design

**Figure 1:**
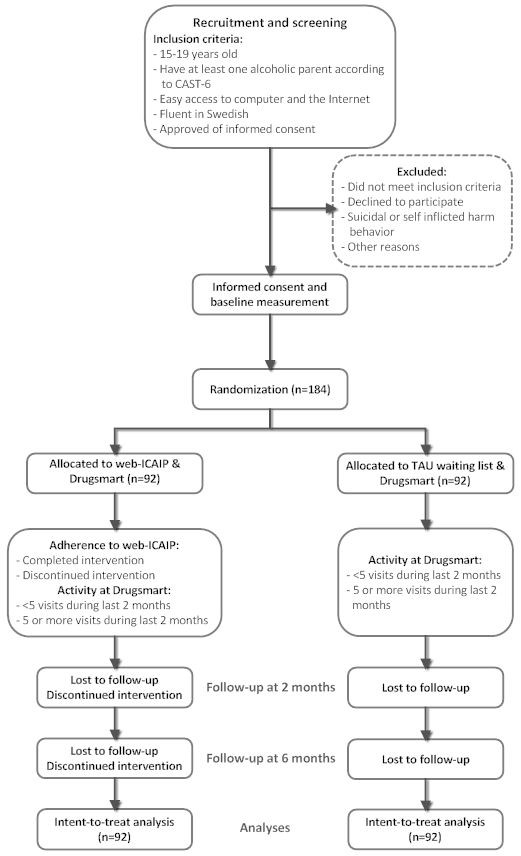
**Flow chart representing the web-ICAIP study design**.

This study consists of a RCT with two parallel conditions (Figure 1) where one group has access to the web-ICAIP intervention and the other consists of a waiting list control group representing treatment as usual (TAU). The intervention is provided *via *the web portal Drugsmart (http://www.drugsmart.com) which, in addition to more general information about alcohol and other drugs, contains information, facts, and activities targeted to children of substance abusing parents. This information has been available at Drugsmart since 2009 and both the intervention group and the control group will have access to this web site.

### The target sample

The sample is to consist of at least 183 adolescents aged between 15 and 19 years. Additional inclusion criteria involve having at least one parent with alcohol problems, easy access to a computer and the Internet, and being sufficiently fluent in Swedish. Participants will be excluded from the study, and referred to appropriate care, if there are indications of either suicidal or self inflicted harm behavior.

### Screening and recruitment

Participants will be recruited through the Drugsmart website and other similar websites by inviting visitors who are 15-19 years old to take part in a web-based survey. Those who agree to participate will access the survey *via *the online survey data collection tool Easyresearch (QuestBack, Norway). Apart from questions concerning age and gender, the participants will be screened for having parents with alcohol problems using the short version of the Children of Alcoholics Screening Test (CAST-6) developed from a 30-item original version [31]. The CAST-6 is a 6-item true/false measure designed to assess whether or not participants perceive their parents' alcohol consumption as problematic, and has proven to be a useful brief screen which compares well to other measures [31,32]. The CAST-6 instrument demonstrates a high internal consistency (r = 0.92-0.94) and test-retest reliability (r = 0.94), as well as a high validity as compared to the 30-item version (r = 0.93) using the recommended threshold score of 3 or higher [31,32]. We have previously translated the CAST-6 into Swedish, and modified it to also include step parents' alcohol consumption, to be used in a feasibility study attempting to measure the prevalence of the problem (T.H.E. & H.L. unpublished data).

Those who are eligible to be included in the RCT receive additional information and are asked to participate. It is required that informed consent is given and an e-mail address is provided, which is the only personal identification record collected and our way to communicate with the participants.

### Assessment

A baseline assessment will take place before randomization, and two follow-ups will take place two and six months after the initial assessment. Participants will be invited to each assessment by e-mails and if necessary, up to three reminders will be sent 5, 10, and 15 days after the first invitation. All assessments are online surveys distributed as described above. To enhance response rates, participants will receive an e-mail containing a cinema gift certificate corresponding to 11 Euros as compensation for completing each assessment. If a participant completes all assessments an additional gift certificate will be given. The participant can subsequently receive four cinema gift certificates totaling 44 Euros.

### Outcome measurements

#### Primary outcomes

To measure depressive symptoms, the child version of the Center for Epidemiological Studies Depression Scale (CES-DC) will be used [33]. This scale measures depressive symptoms during the past week using four-point likert scales, where a higher total score indicate more depressive symptoms. Additionally, this scale has been shown to be a rather general measure of childhood psychopathology [34], and has been demonstrated to be reliable and valid among Swedish adolescents [35].

A coping behavior scale for children of alcoholics has previously been developed at the unit of Clinical Alcohol Research. This scale is based on the Coping Behavior Scale by Orford and co-workers [36] and has been demonstrated to be reliable [27,28]. For the purpose of this study, this scale has been factor analyzed to reduce the number of questions from 37 to 20. The scale measures four coping typologies (discord/emotion, control, relationship, and avoidance) using four-point likert scales, and a threshold score above 50 points (out of 80) indicate a dysfunctional coping behavior.

Alcohol consumption will be assessed using the short version of the Alcohol Use Disorders Identification Test (AUDIT-C) [37], assessing frequency of drinking, the quantity consumed at a typical occasion, and the frequency of heavy episodic drinking. These questions have previously been translated into Swedish [38]. Furthermore, two additional questions will be added concerning whether or not the respondents have ever consumed alcohol to the point that he/she felt intoxicated, and respondents' age of onset of drinking and intoxication.

#### Additional outcomes

Overall life satisfaction will be measured by asking about the participants' past, present, and future rating of his/her life on a ten-point "Ladder of life" representing life status from "worst" to "best" possible life imaginable [39]. The original version was designed for adults and asked the respondents to reflect over the past, present, and future in a five-year perspective. A modified version for children, using a shorter time-frame of one year, has been used previously in Sweden [40] and will be used in this study.

In addition, information about demographics and any previous or present participation in support groups for children of alcoholics will be assessed. Finally, program adherence will be measured in terms of completed film-based lectures and exercises (see below), and respondents' activity at the Drugsmart website will be assessed.

### Randomization

After completing the baseline assessment, each participant will be allocated to the intervention or the control group. An unrestricted random allocation sequence will be generated by an external researcher using the Random Allocation Software [41]. Participants will be informed about their allocation by e-mail, and those who are randomized to the treatment group receive a username and password in order to be able to login to the web-ICAIP website. All participants are informed that they have access to all the other content on the Drugsmart website.

### The intervention

The web-ICAIP is derived from the manual-based face-to-face ICAIP intervention program [27,28]. The ICAIP consists of a combination of an alcohol intervention program, which follows the short version of the Brief Alcohol Screening and Intervention for College Students program (BASICS) [42] and a coping intervention program, and was elaborated in the research group of Clinical Alcohol Research. The program was evaluated in a RCT on adult children of alcoholics who were studying at university level during 2000-2001 [27,28].

Similar to the original ICAIP intervention, the web-ICAIP is divided into an alcohol and a coping theme, consisting of film-based lectures and stories, various exercises, and personalized feedback. Briefly, once the participants have logged into the website they are introduced to the program followed by three film-based lectures (between 8 and 15 minutes each) concerning alcohol problems within the family. There are two additional film-based lectures about alcohol which is optional and aimed at participants with risky alcohol consumption. After completing the lectures, the participants have to go through some exercises concerning alcohol and coping, and thereafter receive a pre-written automatic feedback message. This is followed by some brief information about coping patterns in families of alcoholics as well as emotion- and problem-focused coping. The participants then have to go through and read four so called "readers' letters" which relates to coping and alcohol problems in the family and are presented by film-based introductions each being a few minutes long. Based on these stories, the participants are requested to complete a coping-focused exercise and finally, to reflect over their own family situation.

After a few days, the participants receive an automatic personalized feedback. This feedback compose a library covering all pre-written feedback messages, each one being tailored towards the participants' specific responses during the exercises. The feedback also includes suggestions from experts (*i.e*., therapists who were involved in the development of the intervention) on how to cope and act in the future. In the last part of the intervention the participants also formulate their own action plan for the future. The participants can enter and exit the web-ICAIP anytime during the study period but only receives the personalized feedback once. Since it is recommended that a few days pass in between the different lectures etc., the total estimated time for completing the program is between one and two weeks.

### The control

The control condition consists of a waiting list with unrestricted access to TAU. In Sweden, usual care often involves participating in a support group for children of substance abusing parents, frequently provided by the municipalities. Furthermore, there are a few different Swedish websites available providing information and some level of support to this target group. The one with the greatest focus on alcohol within families is the aforementioned Drugsmart website provided by the Swedish Council for Information on Alcohol and Other Drugs. This website contains general information and facts about alcohol and drugs in addition to more specific information and activities for our target group (*e.g*., a live-chat, a "movie-maker", stories). We have reasoned that it is most ethical sound to suggest that the participants in this study, including those allocated to the control condition, visit the Drugsmart website.

### Sample size

This trial is designed to detect a medium or larger effect size, corresponding to a standardized mean difference (Cohen's d) of > 0.5 [43]. An *a priori *calculation of the estimated sample size, using the software G*Power [44] reveals that it is required that a total of 128 participants (64 in each group) enroll in the trial (power = 0.80, α = 0.05, 2-tailed). However, in a pilot study of a web-based intervention aimed at young people having at least one parent diagnosed with a psychiatric or multiple psychological disorders, or an addiction problem, the attrition rate was found to be 28% [26]. Assuming a similar attrition rate of 30%, we therefore need to enroll a minimum of 128/(1-0.3) = 183 participants in the trial.

### Analyses

In addition to per protocol analysis, data will be analyzed according to the intention-to-treat principle, and all randomized participants will subsequently be included irrespective of whether or not they stayed in the trial. Missing data will be handled by applying the technique of multiple imputation using the Missing Value Analysis routine in the SPSS software (IBM SPSS Statistics 20, IBM Corporation).

Data analyses consist of comparing outcome measurements, at the baseline and subsequent follow-up assessments, within groups and between groups. The effects of the web-ICAIP will be estimated using Cohen's d where a value of around 0.2 indicates a small effect size, and values around 0.5 and 0.8 indicate a medium and large effect size, respectively [43].

### Ethics

This study has been approved by The Regional Ethical Review Board at the Karolinska Institutet (registration nr. 2011/1648-31/5).

## Discussion

The study described herein involves the web-ICAIP, a therapist assisted web-based self-management intervention targeted at adolescents aged between 15 and 19 years, having at least one parent with alcohol problems. The efficacy of this intervention program will be assessed using a RCT study design with two conditions, where one group have access to the web-ICAIP and the other group consists of a waiting list control group. The two experimental conditions will be compared with each other.

### Strengths and limitations

The present study has a number of strengths. First, the web-ICAIP is a web-based intervention program, and it appears as if the Internet is a particularly promising way to provide support to adolescents growing up with parents having alcohol problems, since it offers an anonymous means of communicating and makes intervention programs readily accessible [22]. Additionally, this program is one of the first web-based interventions aimed at this target group and may prove to be an effective strategy to deliver, not only therapist assisted self-management programs, but also other forms of support such as online support groups. Another strength is that the web-ICAIP involves personalized tailored feedback, in the form of pre-written automatic messages and therapist-written personalized feedback, both of which has proven to be important components in web-based interventions aimed at adolescents [45]. Finally, this study attempts to evaluate the efficacy of web-ICAIP using a RCT study design, which is considered to be the strongest experimental design with regards to allocation bias, thus increasing the probability that participant characteristics in the two conditions are similar.

There are also possible limitations to this study. First, the web-ICAIP will be delivered *via *the Drugsmart web portal, which has existed for more than 10 years and since 2009 also includes information and various activities for children growing up with substance abusing parents. This precludes closing down this website during the study period and both the intervention group and the waiting list control group will subsequently have access to Drugsmart. Although the effects of Drugsmart have not been evaluated, it is possible that visitors may be affected, thus threatening the internal validity. We will therefore include questions in the follow-up assessment about the participants' activities on this website. A further limitation is that the participants will be screened for having parents with alcohol problems using the CAST-6 instrument which has not been validated in a Swedish setting. Yet another limitation concerns selection bias and the external validity. Our intention is to recruit study participants *via *Drugsmart and other related websites. It is therefore possible that the majority of the study population can be classified as "information-seeking" adolescents, who may have different personality traits relative the general population. In addition, since an inclusion criterion is to have readily accessibility to a computer and the Internet, it is possible that study participants belonging to a lower socioeconomic class will be underrepresented. Finally, this study is powered to detect a medium effect size. However, if the anticipated effect size is too large the study is under-powered. Similarly, the study may also be under-powered if the attrition rate turns out to be larger than 30%, which is the figure that has been accounted for in our theoretical sample size calculation.

### Implications for practice

Although growing up with parents having alcohol problems *per se *does not predict the development of psychosocial disorders, many of these children are in need for some kind of support. It is therefore discouraging that it is difficult to recruit children to support groups. Not least in Sweden where not even 2% of all children growing up with parental alcohol problems attend to face-to-face support groups provided by the municipalities.

Offering support *via *web-based intervention programs seems particularly appealing for adolescents having parents with alcohol problems. To date, the evidence of such programs is scarce and there is an urgent need for developing and evaluating web-based intervention programs targeting this group of adolescents. This study therefore makes an important contribution to this novel field of research. The results will also provide more insight about effective strategies for the delivering of intervention programs to children of substance abusing parents, and our findings may suggest that other means of support such as online support groups may be delivered *via *the Internet. Finally, this study will add to the growing evidence of the effects of internet-delivered interventions in general.

Depending on the results of this study, the web-ICAIP may become freely available to the public by making the program permanent on the Drugsmart web portal. This website has a large number of visitors (during 2009 and 2010, the mean number of monthly unique visitors was about 10 000 (T.H.E. & H.L. unpublished data) which is considerable given the total size of the Swedish population), thus ensuring the possibility of widespread dissemination.

## Competing interests

HH, NK, and UZ developed the web-ICAIP intervention and the Swedish Council for Information on Alcohol and Other Drugs (CAN) is the owner of the intervention and the Drugsmart website. However, the parties derive no direct financial income from either the web-ICAIP or the Drugsmart website.

## Authors' contributions

THE, NK, and HL obtained funding for this study. HH, NK, and UZ developed the intervention. THE and HL designed this study. HH and UZ contributed to the design of this study. THE wrote this paper. All authors read and approved the final version of this manuscript.

## Pre-publication history

The pre-publication history for this paper can be accessed here:

http://www.biomedcentral.com/1471-2458/12/35/prepub
